# Tissue Composition of *Agave americana* L. Yields Greater Carbohydrates From Enzymatic Hydrolysis Than Advanced Bioenergy Crops

**DOI:** 10.3389/fpls.2020.00654

**Published:** 2020-06-11

**Authors:** Alexander M. Jones, Yadi Zhou, Michael A. Held, Sarah C. Davis

**Affiliations:** ^1^Voinovich School of Leadership and Public Affairs, Ohio University, Athens, OH, United States; ^2^Department of Chemistry and Biochemistry, Ohio University, Athens, OH, United States; ^3^Department of Environmental and Plant Biology, Ohio University, Athens, OH, United States

**Keywords:** CAM, energy, bioethanol, sorghum, switchgrass, miscanthus, crassulacean acid metabolism, biofuel

## Abstract

*Agave americana* L. is a highly productive, drought-tolerant species being investigated as a feedstock for biofuel production. Some *Agave* spp. yield crop biomass in semi-arid conditions that are comparable to C_3_ and C_4_ crops grown in areas with high rainfall. This study evaluates the bioethanol yield potential of *A. americana* by (1) examining the relationship between water use efficiency (WUE) and plant carbohydrates, (2) quantifying the carbohydrate and energy content of the plant tissue, and (3) comparing the products of enzymatic hydrolysis to that of other candidate feedstocks (*Miscanthus x giganteus* Greef et Deuter, *Sorghum bicolor* (L.) Moench, and *Panicum virgatum* L.). Results indicate that (1) WUE does not significantly affect soluble and insoluble (i.e., structural) carbohydrate composition per unit mass in *A. americana*; (2) without pretreatment, *A. americana* biomass had the lowest gross heat of combustion, or higher heating/calorific value, compared to high yielding C_4_ crops; and (3) after separation of soluble carbohydrates, *A. americana* cellulosic biomass was most easily hydrolyzed by enzymes with greater sugar yield per unit mass compared to the other biomass feedstocks. These results indicate that *A. americana* can produce substantial yields of soluble carbohydrates with minimal water inputs required for cultivation, and fiber portions of the crop can be readily deconstructed by cellulolytic enzymes for subsequent biochemical fermentation.

## Introduction

*Agave americana* has the potential to produce commercially viable biomass yields in semi-arid climates (Davis et al., 2011, 2014, 2016). *Agave* species use Crassulacean Acid Metabolism (CAM), a photosynthetic pathway that is associated with both greater water use efficiency (WUE) and greater soluble carbohydrate concentrations in leaf tissue compared to most C_4_ and C_3_ plants (Nobel, 1991; Borland et al., 2009; Davis et al., 2014). The CAM photosynthetic pathway also has greater theoretical maximum energy conversion efficiency than other photosynthetic pathways (Davis et al., 2014). This study evaluates the tissue composition of an obligate CAM species, *A. americana*, grown as a field crop with variable water inputs, and directly compares the tissue composition, hydrolytic degradation, and products of enzymatic reactions with those of high-yielding C_4_ biomass crops.

Advanced lignocellulosic biofuel is produced from either crop residues or stem and leaf biomass of dedicated crops (*i.e*., structural polysaccharides from plant cell walls), unlike first generation biofuels that are exclusively produced from water soluble saccharides (WSS) in grains or seeds (DOE, 2006; Hess et al., 2007; Epa, 2013, 2015; Stock, 2015). In the case of maize, theoretical dry biomass of stalks (stover) have nearly double the yield of grain biomass and a carbon content per unit mass that is similar to grain (Latshaw and Miller, 1924). Although the first advanced lignocellulosic biorefineries are using maize stover as feedstock due to the abundance of this crop, there are alternative feedstock crops that have greater yields and lower input requirements (e.g., tillage, fertilizer, water, pest management) for cultivation (Somerville et al., 2010).

Advanced biofuel crops, including *Agave* spp., *Miscanthus* x. *giganteus* Greef et Deu. (miscanthus), *Opuntia ficus-indica* (L.) Mill., *Panicum virgatum* (L.) Moench (switchgrass), and *Sorghum bicolor* L. (sorghum) have the potential to reduce greenhouse gas (GHG) emissions associated with biofuel production (Davis et al., 2009, 2013; Somerville et al., 2010; Yan et al., 2011; Cushman et al., 2015). Harvestable dry biomass for *A. americana* is projected to yield up to 9.3 Mg ha^–1^ y^–1^ in semi-arid conditions (Davis et al., 2016). Average biomass yields from the perennial grasses miscanthus and switchgrass are ca. 23.4 Mg ha^–1^ y^–1^ and 10.0 Mg ha^–1^ y^–1^, respectively (Arundale et al., 2015), while yields of sorghum biomass are 22.0 Mg ha^–1^ y^–1^ on average (Grennell, 2014). Both perennial grasses require less fertilizer compared to conventional agricultural crops because of the ability to efficiently recycle nitrogen (Christian et al., 2006; Heaton et al., 2009; Davis et al., 2010) and the root systems of perennial species allow for greater carbon sequestration compared to annual species (Sartori et al., 2006; Heaton et al., 2009; Davis et al., 2010).

Plants with CAM have the greatest theoretical WUE, and in some cases species have high annual productivity coupled with high concentrations of soluble carbohydrates stored in plant tissue (Nobel, 1990, 1991; Borland et al., 2009; Davis et al., 2011, 2019). Recent research emphasizes the potential for CAM species, such as *Agave* spp. and *Opuntia* spp. (prickly pear), to be grown as biofuel feedstock on marginal or arid lands that are deemed unsuitable for food crops or pasture lands (Smith, 2008; Borland et al., 2009; Chambers et al., 2010; Davis et al., 2011, 2019; Li et al., 2014; Stewart, 2015; Yang et al., 2015; Cushman et al., 2015). *Agave americana* has not been grown in commercial agriculture in the past, but the first field trials and models of productivity indicate it is a viable feedstock crop in semi-arid conditions (Davis et al., 2016; Niechayev et al., 2018). Here, we assess the difference in plant tissue composition of *A. americana* grown with different water inputs to determine if soluble carbohydrates vary as WUE increases in drier conditions.

In addition to potentially high concentrations of WSS, *A. americana* also has lignocellulosic biomass that may be useful in fuel production. In advanced bioethanol production that makes use of lignocellulosic biomass, lignin must be separated from cellulose and hemicellulose to make the carbohydrates available for hydrolysis (Moorhead and Reynolds, 1993; Roche et al., 2009; Humbird et al., 2011); this can be accomplished biologically using specialized enzymes (Evans et al., 1994). Aromatic substances within the primary wall may act as nucleation sites for lignin biosynthesis and may act to cross-link lignin polymers and other polysaccharides to pectin, glycoprotein and/or hemicellulosic matrices. Aromatic residues, such as ferulic acid, are present adjacent to arabinose units of type II xylans and some glucose and arabinose units of pectic polysaccharides (Markwalder and Neukom, 1976; Grabber et al., 2000; Lam et al., 2001), and this effect can increase biomass recalcitrance to enzymatic hydrolysis and exacerbate the persistence of inhibitors (Kim et al., 2011; Jönsson et al., 2013; Jönsson and Martín, 2016).

Both the plant tissue composition and hydrolytic degradation of *A. americana* are evaluated in this study and compared to three advanced biofuel feedstocks that use the C_4_ photosynthetic pathway (i.e., miscanthus, switchgrass, and sorghum). Previous work to increase fermentable products from lignocellulosic biomass includes improving pretreatment methods to increase enzymatic hydrolysis (Faik, 2013; Camesasca et al., 2015; Davis R. et al., 2015; Jin et al., 2016), biochemical engineering to improve enzyme activity (Zhang et al., 2006; Raghuwanshi et al., 2014), discovering novel cellulolytic enzymes (Zhang et al., 2006; Rani et al., 2014), optimizing enzyme blends for specific feedstocks (Gao et al., 2010), and decreasing the cost of enzymes (Klein-Marcuschamer et al., 2012; Culbertson et al., 2013; Johnson, 2016). This study specifically evaluated acid hydrolysis, enzymatic hydrolysis with a commercially available enzyme mixture, and saccharification of plant tissue from each of the four feedstocks to quantify the difference in conversion efficiency with difference tissue compositions.

Gross heat of combustion (GH), also referred to as the calorific value or the Higher Heating Value (HHV), relates to biofuel yield because it is negatively correlated with enzymatic digestion efficiency of biomass, and high GH values are associated with high lignin and mineral contents (Demirbaş, 2001; Sheng and Azevedo, 2005; Godin et al., 2013). The energy and chemical inputs required for separating and degrading cellulose in *Agave* spp. may be reduced because of lower lignin concentrations relative to other feedstock crops (Davis et al., 2011). Little research has thus far described the lignocellulosic conversion of *A. americana* and how it directly compares to other lignocellulosic feedstocks of interest (Saucedo-Luna et al., 2011; Corbin et al., 2015; Mielenz et al., 2015; Yang et al., 2015; Pérez-Pimienta et al., 2018; Wang et al., 2019).

This study evaluates the potential bioethanol yield from *A. americana* by resolvingGH was adjusted for moistur e(1) carbohydrate concentrations, (2) overall differences in plant tissue composition relative to other advanced biofuel feedstocks, and (3) enzymatic conversion efficiencies of cellulose and hemicellulose. The potential energy yield of *A. americana*, a vigorous CAM plant, is also compared with the potential energy yield of high-yielding C_4_ crop species (miscanthus, switchgrass, and sorghum).

## Materials and Methods

Plant tissue compositional analysis, energy content analysis, and enzymatic hydrolysis were completed to determine differences in biomass quality and hydrolytic conversion efficiency of *A. americana* relative to other advanced biofuel crops. Plant tissue samples from two field sites, one with an *A. americana* crop and the other with switchgrass, miscanthus, and sorghum crops, were analyzed using identical Laboratory Analytical Procedures (LAP) developed by the National Renewable Energy Laboratory (NREL) to determine the percentage of carbohydrates within biomass. Carbohydrate concentrations of *A. americana* grown in a field experiment with different irrigation treatments were resolved, compared, and then regressed against the WUE associated with different water inputs, as determined in previous work (Davis et al., 2016). Both GH and sugar yield from enzymatic hydrolysis were measured in all four feedstocks and compared. Detailed methodologies are provided in the following sections.

### Field Characteristics for *A. americana*

A 3-acre site, located at the University of Arizona Maricopa Agricultural Center (elevation, 363.77 m; 33° 05′ N, 111° 97′ W), was divided into eight plots, each containing six 15 m^2^ subplots (Davis et al., 2016). In 2012, forty-nine individual *A. americana* were planted in two randomly selected subplots within each plot; individuals were spaced 2 m apart, within and along rows. The two planted subplots in each plot were designated as one of four different irrigation treatments for the duration of the experiment (4 years). The plots were flood irrigated at different levels (100, 260, 330, and 580 mm y^–1^), and the field received ∼200 mm mean annual precipitation over four years (2012–2016). Mean annual water inputs (MWI) for the treatments (including both irrigation and precipitation) totaled 300, 460, 530, and 780 mm y^–1^, respectively. The soils at this site are characterized as sandy clay loam. For more information pertaining to field conditions at this site, see Davis et al. (2016). Samples used in this study were harvested and dried in the oven as per Hames et al. (2008a) in January 2016 and stored in paper bags at room temperature in a dry, dark place until analysis was accomplished.

### Field Characteristics for Temperate Grasses

Experimental plots of advanced cellulosic bioenergy crops were established at the Ridges Land Lab at Ohio University (elevation 222 m; 39° 19′ N, 82° 06′ W) on abandoned agricultural land; one site (site “A”) was historically used for pasture, and the other site (site “B”) was historically managed for hay. Soils in this area are mostly Aquic Hapludalfs and Typic Hapludalfs, and the land was unmanaged from 1992 until the time this experiment was established in 2013. Three replicate 10 m × 10 m plots for each of the advanced biofuel feedstock species were randomly assigned within each site with 2 m spacing between plots that were mowed regularly (Supplementary Figure 1).

Miscanthus rhizomes were donated by Tom Voigt from the University of Illinois at Urbana-Champaign (UIUC), and planted May 8, 2013. Sorghum seed (CHR-FS4; Chromatin; New Deal, TX, United States) was donated by Dr. Pat Brown from UIUC, planted in rows during the first week of June spaced 1 m apart and seeds within a row spaced 5 cm, and fertilized the second week of June (2013, 2014, and 2015). Switchgrass seed (‘Timber’ var.) was sourced from Ernst Conservation Seed and planted April 25, 2013. Sorghum was harvested in November 2015 following senescence, and air-dried for 6 weeks. Switchgrass and miscanthus were harvested in February 2016 following senescence; above-ground biomass was harvested, bundled and air-dried in the field for 4 weeks as would be expected in biomass crop management. Subsamples were oven dried for tissue composition analysis as described in subsequent sections.

### Analysis of GH

Gross heat of combustion was measured using a Parr 6200EA isoperibol (oxygen bomb) calorimeter (Parr Instrument Company; Moline, IL, United States) in conjunction with a Parr 6510 water handling system to maintain constant temperature (Parr Instrument Company) per method ASTM D5865. Benzoic acid (GH = 6318 cal g^–1^) was used as a standard for calibration. GH was adjusted for moisture content (GH_OD_) using equation 1 (Boundy et al., 2011).

(1) $$G\,H_{O\,D}=\frac{G\,H_{s\,a\,m\,p\,l\,e}}{\left(\frac{\%TotalSolids}{100}\right)}$$

The GH value was converted to MJ kg^–1^ from cal g^–1^ using 0.004187 as a conversion factor. Measured GH of samples was compared to theoretical values calculated from lignin contents using equations 2a and 2b from Demirbaş (2001).

(2a) $$GH_{1}=0.0889\left(\%L\right)+16.8218\;$$

(2b) $$GH_{2}=0.0877\left(\%L\right)+16.4951\;$$

where GH_n_ is the theoretical GH value (MJ kg^–1^) and %L is percent lignin. The percent lignin was measured according to methods described in section “Determination of Lignin.”

### Composition Analysis

**FIGURE 1:**
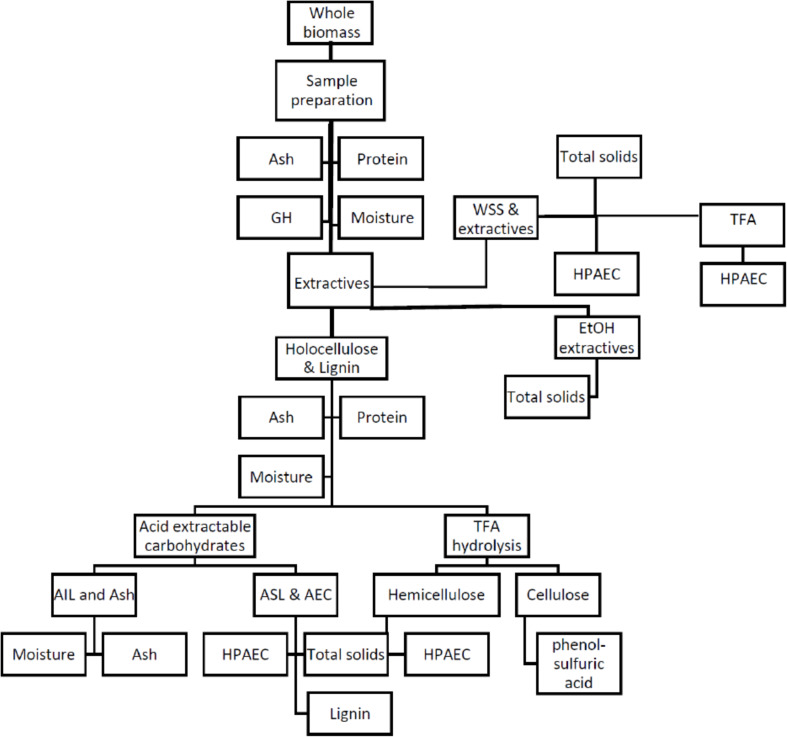
Flowchart outlining the composition analysis procedures for *A. americana* adapted from Sluiter and Sluiter (2011). Whole biomass sample is prepared, extracted with water to remove water-soluble carbohydrates (WSS), extracted with ethanol to remove inhibitors from residue containing holocellulose and lignin, which is then hydrolyzed to solubilize some lignin (ASL) and acid extractable carbohydrates (AEC) (*i.e*., structural carbohydrates) and leave a residue containing insoluble lignin (AIL) and minerals. Water soluble extract and holocellulose fraction undergo TFA hydrolysis and analyzed by HPAEC, and TFA insoluble is analyzed for cellulose content by phenol-sulfuric acid assay. Lignocellulosic grasses were only be analyzed for GH, WSS, and AEC. Procedure short titles are provided in non-shaded boxes, and biomass fraction name is provided in shaded ovals.

Composition analysis of field grown *A. americana* was performed per LAPs developed by NREL and Moorhead and Reynolds (1993) wherein sequential fractionation was used to determine extractives, minerals, cellulose, lignin, and ash contents. The review by Sluiter and Sluiter (2011) provides mass closure for the LAPs published by NREL, and the herbaceous feedstocks calculation spreadsheet published by NREL was used in this work. A workflow is given in Figure 1. Two randomly selected replicate samples were analyzed from each subplot (*n*_subplot_ = 1), which equates to eight replicates per irrigation treatment (*n*_treatment_ = 4), and a total *N* = 16 for all four treatments. In the case of the C_4_ grass feedstocks, WSS and acid-extractable carbohydrates were analyzed per NREL protocols for comparison with *Agave*, and then also analyzed for GH.

#### Tissue Sample Preparation

Leaves from *A. americana* were harvested from the plants at the end of January 2016, dried in an oven as per Hames et al. (2008a), then shipped to Ohio University. Plant tissue samples from the grass species were collected following field drying, and oven-dried to remove residual moisture that remained following field drying. Oven dried samples were stored in paper bags at ambient room temperature and processed as per Hames et al. (2008a). In summary, samples were ground to uniform particle size using a Wiley mill (Thomas Scientific, 3383L10, United States) with a 20 mesh (0.85 mm) screen. Once samples were ground, they were kept in 3.79 L airtight polyethylene zipper storage bags at 2°C.

#### Determination of % Total Solids

Total solids and moisture present in biomass and liquid samples was determined gravimetrically from methods from Sluiter et al. (2008a). Total solids were calculated as a percentage of mass (%), given in equation 3.

(3) $$\%TotalSolids=\frac{M_{d\,r\,y}}{M_{w\,e\,t}}\times 100\;$$

#### Determination of Protein Content

Protein content was determined by quantifying percent N via combustion and calculated using an appropriate N factor for the plant amino acid profile. Percent N content of biomass was determined by combustion on a Costech ECS 4010 (Costech Analytical Technologies, Inc., Valencia, CA, United States). Samples (5.0 ± 0.5 mg and 7.5 ± 0.5 mg for whole biomass and extractive-free biomass (EFBM), respectively) were combusted and detected in the gas phase using a thermal conductivity detector and a Colibrick A/D transducer (U32; DataApex→ Ltd., Prague, Czechia) with Clarity→ Elemental Analysis Software (C50; DataApex→ Ltd.). The measured %N was used to calculate percent protein with a protein factor (PF) of 4.6, as determined suitable for biomass without a characterized amino acid profile by Hames et al. (2008b), using equation 4.

(4) %Protein=PF×%N              

Protein content was determined in whole biomass to calculate whole protein content in the sample and EFBM, and was not determined in the post-hydrolysis solid residue. It was assumed that minimal protein co-condensation occurred within the post-hydrolysis residue because dried hydrolysate had very low %N (as observed when determining acid-soluble lignin).

#### Determination of Ash

Percent ash in biomass was determined by methods that were adapted from the LAP by Sluiter et al. (2008b). Ash content was measured in whole biomass, EFBM, and the post-hydrolysis residue. Percent ash was estimated based on oven dry mass of the sample (M_dry_), as follows:

(5) $$\%Ash=\frac{\left(M_{c\,r\,u\,c\,i\,b\,l\,e+a\,s\,h}-M_{c\,r\,u\,c\,i\,b\,l\,e}\right)}{M_{d\,r\,y}}\times 100\;$$

#### Determination of Water-Soluble Extractives and Carbohydrates

A water extraction was performed to remove WSS and other water-soluble compounds by adding 15 mg ± 2.5 mg biomass (M_Whole BM_) to preweighed 25 mL centrifuge tubes (W_tube_). 12.5 mL distilled water (dH_2_O) was added and the tube was sealed and placed into a water bath at 60°C for 30 min. The mixture was centrifuged (∼125 × *g*) for 15 min and the supernatant collected and stored. Two more water extractions were performed and combined for a total of 37.5 mL extract. A subsample (5 mL) was used for determination of total dissolved solids in the water extract.

Water soluble saccharides were quantitatively determined from a 0.75 ml subsample. Samples were centrifuged and dried under a steady stream of air. Dried extracts were dissolved in 0.75 mL cellobiose (20 mM in dH_2_O) (C7257; Sigma-Aldrich Co., LLC.), filtered through 0.22 μm nylon spin filters (8169; Corning Inc., Corning, NY, United States), and appropriately diluted (1:1000 and 1:100 for *A. americana* and grasses, respectively) with de-ionized water. Aliquots (25 μL) were fractionated by high-pH anion-exchange chromatography with pulsed amperometric detection (HPAEC-PAD) (Dionex Corp., Sunnyvale, CA, United States) fitted with a Carbo-Pac PA20 anion-exchange column (Dionex Corp.) and similar guard column (50 × 4 mm i.d.; Dionex Corp.). Elution proceeded as an isocratic flow (0.5 ml min^–1^, for all samples) in 40 mM sodium hydroxide (NaOH) for 4 min, followed by a linear increase to 40 mM NaOH and 40 mM sodium acetate over 5 min. Peak areas of the fractionated sugars were integrated and quantified by molar response factors generated from the peak areas measured for sugar standards D-(+)-glucose (Glc) (G8270; Sigma-Aldrich Co., LLC.; St. Louis, MO, United States), D-(−)-fructose (Fru) (F0127; Sigma-Aldrich Co., LLC.), and sucrose (Suc) (S7903; BioXtra; Sigma-Aldrich Co., LLC.) using cellobiose as an internal standard (0.5 nmol, each).

#### Determination of Ethanol-Soluble Extractives

Removal of extractives to achieve EFBM was necessary to avoid inhibitors that can negatively impact biomass analysis and acid hydrolysis of structural oligomeric carbohydrates (Thammasouk et al., 1997; Sluiter et al., 2008c). Samples were extracted from plant tissues using a method adapted from Moorhead & Reynolds (1993).

Percent extractives were calculated from equation 6.

(6) $$\%Extractives=\frac{M_{W\,h\,o\,l\,e\,B\,M}-\left(M_{E\,F\,B\,M+t\,u\,b\,e}-M_{t\,u\,b\,e}\right)}{W_{W\,h\,o\,l\,e\,B\,M}}\times 100\;$$

where M_Whole BM_ is the oven dry weight of whole biomass and M_tube_ is the mass of the preweighed tube.

#### Determination of Acid-Extractable (Structural) Carbohydrates

An acid hydrolysis using sulfuric acid (H_2_SO_4_) was performed to solubilize structural carbohydrates and acid soluble lignin and separate these components from the residue containing insoluble lignin and structural inorganic material. Dry EFBM (0.50g) were hydrolyzed using methods adapted from Moorhead and Reynolds (1993) and Sluiter and Hames (2012). Oven dry EFBM mass (ODM_sample_) was calculated from equation 7.

(7) $$O\,D\,M_{s\,a\,m\,p\,l\,e}=\frac{M_{E\,F\,B\,M}\times \%TS}{100}\;$$

where % total solids was calculated using equation 3.

Acid insoluble residue (AIR) was calculated by equation 8:

(8) $$\%AIR=\frac{M_{f\,i\,l\,t\,e\,r+s\,a\,m\,p\,l\,e\,\left(H\,B\,M\right)}-M_{f\,i\,l\,t\,e\,r}}{O\,D\,M_{s\,a\,m\,p\,l\,e}}\times 100\;$$

AIR was prepared from *A. americana* and the grasses following Held et al. (2011) to determine composition of hemicellulose. Hemicellulose sugar compositions were determined on AIR from *A. americana* and grass samples as per York et al. (1986). AIR samples (25 μg) and standards (100 nmol) were hydrolyzed in 2N trifluoroacetic acid (TFA) at 121°C for 90 min. Samples were cooled to room temperature then centrifuged (3000 × *g*) for 3 min. TFA was evaporated under a steady stream of nitrogen at 45°C. All samples were then dissolved again in 500 μL deionized water, filtered using Corning Costar Spin-X centrifuge tube filters (CLS8160), and then stored at −20°C. Aliquots (25 μL) were fractionated by HPAEC (Dionex Corp., Sunnyvale, CA, United States) fitted with a Carbo-Pac PA20 anion-exchange column (Dionex Corp.) and similar guard column (50 × 4 mm i.d.; Dionex Corp.) as per Øbro et al. (2004). Peak areas of the fractionated sugars were integrated and quantified by molar response factors generated from the peak areas of external sugar standards (4-point calibrations). Following TFA hydrolysis, an additional phenol-sulfuric acid hydrolysis was performed on the TFA-insoluble pellet as per DuBois et al. (1956) to determine the proportion of cellulose using Glc as a standard.

Additionally, TFA hydrolysis was performed on the water extract of *A. americana* to resolve the amount of oligomeric sugar present as starch and fructans within the liquid. The TFA water extract hydrolysate was analyzed by HPAEC as described above to measure Glc, Fru, and Suc from oligomers.

#### Determination of Lignin

Some lignin was removed from the material during acid extraction, while some remained insoluble within the cell wall matrix. Acid soluble lignin present within the hydrolysate was analyzed within six hours and determined from the concentration of dissolved organic carbon (DOC) (Browning, 1967; Lin and Dence, 1992; Weishaar et al., 2003; Sluiter and Hames, 2012). An aliquot (5 mL) of hydrolysate was used for DOC quantification. Acid soluble lignin was determined by comparing absorbance at 320 nm (A_320_) against a blank containing 4% H_2_SO_4_; for lignin from maize, absorbance is greatest between 260–380 nm with relative maxima at 280 and 320 nm (Müse et al., 1997). ASL was calculated from equation 9:

(9) $$\%ASL=\frac{A_{320}\times V_{f\,i\,l\,t\,r\,a\,t\,e}\times D}{S\,U\,V\,A_{320}\times O\,D\,M_{s\,a\,m\,p\,l\,e}}\times 100\;$$

where A_320_ is the average ultraviolet (UV) absorbance at 320 nm, V_filtrate_ is the hydrolysate volume (86.73 mL), D is a dilution factor if used, SUVA_320_ (L g^–1^ cm^–1^) is the Specific UV Absorbance (SUVA) at 320 nm (SUVA_320_), ODM_sample_ is the mass of the sample (mg) from equation 7. SUVA, also described as the specific absorptivity or the molar absorbance coefficient for a given concentration of dissolved carbon (Browning, 1967; Lin and Dence, 1992), was used to estimate the percent lignin by colorimetrically measuring aromaticity; results of analysis of dissolved organic carbon (DOC) and humic substances by Weishaar et al. (2003) showed 97% correlation between SUVA and ^13^C nuclear magnetic resonance (NMR) values for aromaticity. SUVA_320_ was calculated from equation 10,

(10) $$S\,U\,V\,A_{320}=\frac{A_{320}}{\left[D\,O\,C\right]}\times L_{p\,a\,t\,h}\;$$

where A_320_ is the measured absorbance at 320nm. [DOC] was determined by combustion of dried down hydrolysate on a Costech ECS using the same method as described previously for protein determination, but percent carbon (%C) was used in lieu of %N, and L_path_ was the path length (cm) of the spectrophotometer cell; [DOC] was calculated by drying down 5mL of H_2_SO_4_ hydrolysate at 105°C for 48 h and combusting a sample (5.5 ± 0.5 mg) as previously described, from equation 11,

(11) $$\left[DOC\right]\left(gL^{-1}\right)=\%C\times \left(\frac{M_{s\,a\,m\,p\,l\,e+p\,a\,n}-M_{p\,a\,n}}{V_{s\,a\,m\,p\,l\,e}}\right)\;$$

where %C was determined from combustion, M_pan_ is the mass of the drying pan (g), M_sample+pan_ is the mass of the pan and dried sample (g), and V_sample_ is the volume (L) of liquid sample dried down. Acid insoluble lignin (AIL), or Klason lignin, was calculated using equation 12.

(12) $$\%AIL=\frac{M_{s\,a\,m\,p\,l\,e\,\left(H\,B\,M\right)}-M_{a\,s\,h}}{O\,D\,W_{s\,a\,m\,p\,l\,e}}\times 100\;$$

Percent lignin on an extractives free basis can be calculated using equation 13,

(13) $$\%Lignin_{E\,x\,t.F\,r\,e\,e}=\%AIL+\%ASL\;$$

where %AIL was calculated from equation 12 and %ASL from equation 9. Total lignin, based on whole biomass was calculated from equation 14,

(14) $$\%Lignin_{w\,h\,o\,l\,e}=\left(\%Lignin_{E\,x\,t.F\,r\,e\,e}\right)\times \frac{\left(100-\%Extractives\right)}{100}\;$$

where %Lignin_Ext. Free_ was calculated from equation 13, and %Extractives is from equation 6.

### Comparison of Enzymatic Digestibility

Enzymatic hydrolysis conversion efficiency was evaluated for each of the four feedstocks to determine biomass recalcitrance using purified and lyophilized enzymes. Purified cellulase from *Trichoderma reesei* ATCC 26921 (C8546; Sigma-Aldrich), xylanase from *Trichoderma viride* (X3876; Sigma-Aldrich), and beta-glucosidase from almonds (49290; Sigma-Aldrich) were used for hydrolysis. Prior to comparing enzymatic digestibility of a biomass substrate, protein content of the enzyme solution was determined using a Pierce bicinchoninic acid (BCA) protein quantification kit (BCA1; Sigma-Aldrich Co., LLC.). The BCA quantification assay was recommended for fungus-derived enzymes because cellulase produced by the species *T. reesei* contains roughly 50% cellobiohydrolase I (CBH I; Cel7A), and this cellulolytic enzyme and others within the same glycoside hydrolase family (GHF) 7 may react poorly to other calorimetric assays (Resch et al., 2015). Protein content of the enzyme cocktail determined from the Pierce BCA protein assay was 14.47 mg mL^−1^ ± 1.17 mg mL^−1^ for the mixed enzymes. Three samples from each of the four plant species studied here were subjected to enzymatic hydrolysis treatments using the mixed enzymes.

#### Low Solids Loading Enzymatic Hydrolysis

Enzymatic saccharification of EFBM using low solids loading was performed according to Resch et al. (2015). EFBM was washed three times with 30mM sodium citrate buffer (pH 5.0) containing 0.002% (w v^–1^) sodium azide to remove soluble sugars and inhibitors, and the residue was suspended in the same buffer at a loading rate of 2.0% (w v^–1^). A sample containing 0.014 g EFBM, corrected for moisture, was quantitatively transferred to a screwcap test tube, and 42 μL 1.0 M sodium citrate buffer (pH 5.0) was added. 5.6 μL of 5.0% (w v^–1^) sodium azide was added to each vial to inhibit microbial contamination. The volume of enzyme solution used was calculated from equation 15, so 20.0 mg protein from enzyme was available per g glucan,

(15) $$E\,n\,z\,y\,m\,e\,v\,o\,l\,u\,m\,e=\frac{1.0\,m\,L}{X\,m\,g\,p\,r\,o\,t\,e\,i\,n}\times \frac{20.0\,m\,g\,p\,r\,o\,t\,e\,i\,n}{1.0\,g\,g\,l\,u\,c\,a\,n}\times g\,g\,l\,u\,c\,a\,n\;$$

where X was mg protein as calculated from 1.0mL enzyme solution using BCA kit. Released sugars (*e.g*., cellobiose, Glc, and Xyl) were analyzed using HPAEC, as previously described in the determination of structural carbohydrates.

#### Determination of Liberated Sugars

Resulting sugar concentrations (mg mL^–1^) from each digestion mixture were used to determine percent conversion during hydrolysis as the amount of sugar liberated (i.e., concentration hydrolyzed oligomeric sugar) divided by the initial mass of EFBM, as in equation 16,

(16) $$\%hydrolysis=\frac{\frac{m\,g\,s\,u\,g\,a\,r}{m\,L}\times 14\,m\,L}{0.14\,g\,E\,F\,B\,M}\times 100\;$$

where sugar (mg mL^–1^) is quantified by HPAEC.

### Statistical Analysis

Analysis of Variance (ANOVA) was used to statistically compare tissue composition, GH of biomass, and products of enzymatic digestion from *A. americana* plants that were grown with different MWI, and to compare these characteristics of biomass between the four crop species. *Post hoc* Tukey’s honest significant difference (HSD) was used to resolve significant differences between treatment groups or species with α = 0.05.

## Results

### Composition Analysis

Composition analysis of the feedstocks measured in this study indicated that the leaf tissue of *A. americana* differed substantially from the grass feedstocks (Table 1). *Agave americana* had the largest mean proportion of water soluble saccharides compared to miscanthus, sorghum, and switchgrass (0.25, 1.14, and 0.28%, respectively; *p* = 7.42 × 10^–9^) and much lower hemicellulose (5.63%) relative to miscanthus (24.53%), sorghum (21.83%), and switchgrass (24.22%) (*p* = 1.11 × 10^–16^; Table 1). Cellulose was also significantly different (p = 5.22 × 10^–15^; Table 1), with *A. americana* having the lowest cellulose (5.55%) relative to the other feedstocks, and miscanthus having the greatest cellulose content (32.43%). Total holocellulose contents for the four feedstocks in descending order were 59.96% for miscanthus, 46.73% for switchgrass, 41.50% for sorghum, and 11.18% for *A. americana* (Table 1). The C_4_ crops had similar tissue compositions to one another, and miscanthus and switchgrass were the most similar (Table 1).

**TABLE 1:** Composition analyses from experimental and literature values for dry untreated biomass for *A. americana (Ag), M. x giganteus (Mi), S. bicolor (So)*, and *P. virgatum (Sw)*. Water soluble saccharides (WSS), hemicellulose (Hc), cellulose, holocellulose (HoC), lignin, and carbon (C) are reported as a percentage of dry biomass.

Species	%WSS	%Hc	%Cellulose	%HoC	%Lignin	%C
Ag	43.95*a*	5.63*a*	5.55*a*	11.18*a*	9.06	38.86*a*
Mi	0.25*b*	24.53*b*	32.43*c*	56.96*c*	−	45.59*c*
So	1.14 *b*	21.84*b*	19.66*b*	41.50*b*	−	42.38*b*
Sw	0.28 *b*	24.22*b*	22.51*b*	46.73*b*	−	42.99*b*

*Lowercase italicized letters designate significant differences within a column as resolved from one-way ANOVA and Tukey’s HSD test (*p* < 0.05).*

#### WSS in *A. americana*

**FIGURE 2:**
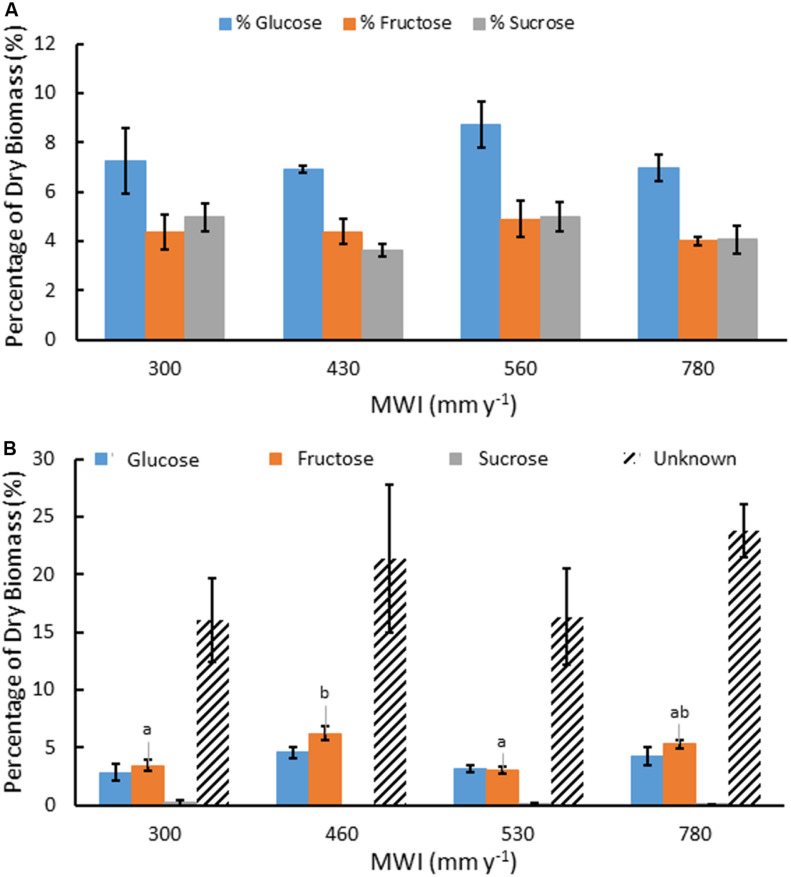
Mean concentration of water soluble Glc, Fru and Suc as a percentage of dry biomass **(A)** and saccharides produced after TFA hydrolysis of water soluble extracts including Glc, Fru, Suc, and unknown sugars from oligosaccharides **(B)**, for *A. americana* under different MWI (mm y^–1^). Error bars indicate standard error of the mean (*n* = 4). Letters indicate significant differences between species in Fru (*p* < 0.05) from one-way ANOVA and Tukey’s HSD test. No significant difference in the other sugars were observed between species (*p* > 0.05).

Water soluble extracts in *A. americana* ranged from 42.21% to 45.37% of dry leaf biomass and contained soluble mono-, di-, and oligosaccharides (Table 2). Water extracts contained 16.3% total mono- and disaccharides Glc, Fru, and Suc in a ratio of 10:6:6, respectively (*n* = 16). There was no significant difference between total mono- and disaccharide content present in the water-extracted fraction from plants grown with different irrigation treatments (*p* = 0.335). Mean percent mono- and disaccharides were 7.46, 4.42, and 4.42% for Glc, Fru, and Suc, respectively (*n* = 16). There was no significant difference for individual monosaccharide contents of the water extract between plants from different irrigation treatments (*p* = 0.435, 0.769, 0.218 for Glc, Fru, and Suc, respectively; Figure 2a).

**TABLE 2:** Mean annual water input (MWI) in mm y^–1^, water-soluble saccharides (WSS) as % of dry biomass, WSS yield (Mg ha^–1^ y^–1^) calculated based on biomass yield reported in Davis et al. (2016), and WSS WUE (WUE_WSS_) expressed as WSS yield per MWI (kg WSS ha^–1^ mm^–1^) for *A. americana*.

MWI^1^ (mm y^–1^)	%WSS	WSS Yield (Mg ha^–1^ y^–1^)	WUE_WSS_ (kg WSS ha^–1^ mm^–1^)
300	42.45 ± 6.08	1.70 ± 0.24*a*	5.66 ± 0.81*ab*
460	46.49 ± 5.69	3.16 ± 0.39*b*	6.87 ± 0.84*ab*
530	46.53 ± 3.67	4.33 ± 0.34*b*	8.17 ± 0.64*a*
780	45.37 ± 4.07	3.31 ± 0.30*b*	4.25 ± 0.38*b*

*Standard error indicated after each mean; *n* = 4. Lowercase italicized letters designate significant differences within a column as resolved from one-way ANOVA and Tukey’s HSD test (*p* < 0.05). Where no letters are indicated, there was no significant difference (*p* > 0.05).*

There was also a large percentage of water soluble material (∼35%) that was not identified as monosaccharide by HPAEC. TFA hydrolysis of the water extract yielded mostly monosaccharides derived from water-soluble oligosaccharides and the unresolved fraction was subjected to a phenol-sulfuric acid assay to determine the unknown oligosaccharides in glucose equivalents. Oligosaccharides present within the water extractable fraction of *A. americana* leaf tissue represented nearly 10% of dry biomass (Figure 2b). There was no significant difference in Glc nor Suc from oligomers across irrigation treatment (*p* = 0.18 and 0.55, respectively). There was a significant difference between Fru from oligomers across differing water inputs (*p* = 0.0008); maximum Fru content was measured in biomass from crops receiving MWI of 460 mm y^–1^ (6.19 ± 0.61%) while the lowest Fru content was 3.06% (± 0.30%) in plants receiving MWI of 530 mm y^–1^.

**FIGURE 3:**
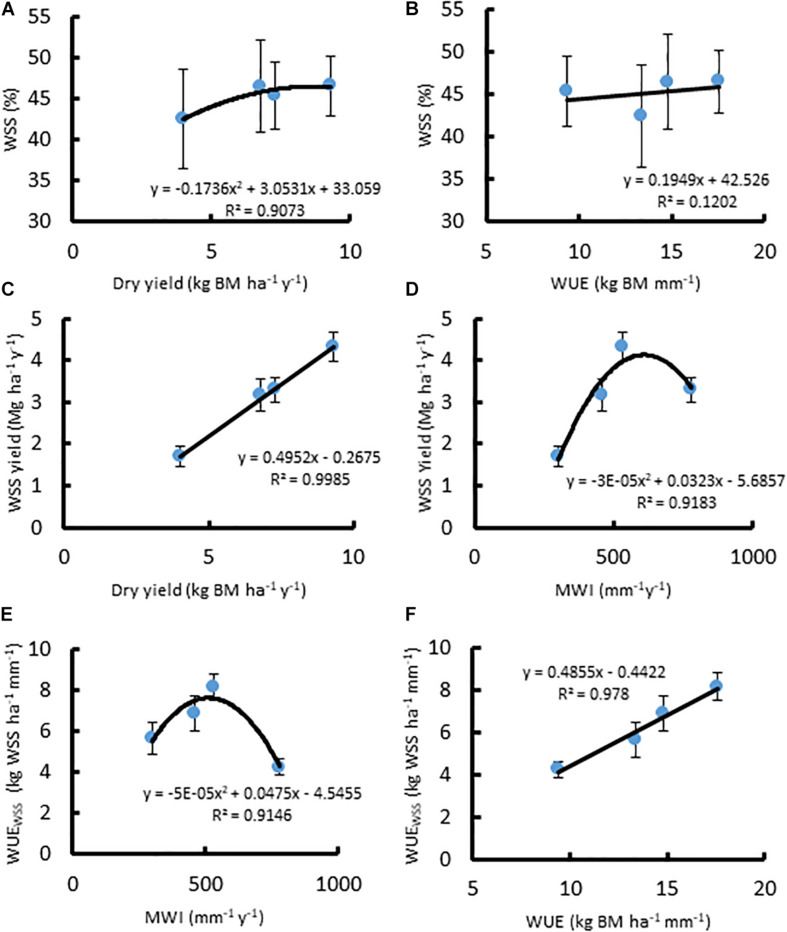
Relationships between **(A)** dry biomass yield (Mg ha^–1^ y^–1^) and WSS (%), **(B)** WUE (kg ha^–1^ mm^–1^) and WSS (%), **(C)** dry biomass yield and WSS yield (Mg ha^–1^ y^–1^), **(D)** MWI (mm y^–1^) and WSS yield, **(E)** MWI and WUE_WSS_ (kg WSS ha^–1^ mm^–1^), and **(F)** WUE and WUE_WSS_, for *A. americana*. Error bars indicate standard error of the mean; *n* = 4.

In *A. americana*, WUE was found to be positively, but weakly, correlated with percent water soluble mono- and disaccharides (*y* = 0.1949x + 42.526, *R*^2^ = 0.12018; Figure 3b). The total percentage of WSS was found to be correlated with dry yield (kg ha^–1^), using a positive second-order polynomial equation (*y* = −0.1736x^2^ + 3.0531x + 33.059; *R*^2^ = 0.90728) (Figure 3a). Water soluble monosaccharide yields were largest in individuals that were grown with 560 mm MWI (Figure 3d). WSS yield was highly correlated (*y* = 0.4952x − 0.2675; *R*^2^ = 0.99848) with dry biomass yield (Figure 3c) because there was no significant difference in the concentration of WSS across irrigation treatment. Similarly, MWI and WSS per unit water (WUE_WSS_; kg WSS ha^–1^ mm^–1^) exhibited a similar trend with a maximum WUE *ca.* 500mm y^–1^ (Figure 3e). There was no significant difference associated with WSS concentrations across irrigation treatment, and there was a strong linear correlation between WUE_WSS_ and WUE (*y* = 0.4855x− 0.4422; *R*^2^ = 0.97796; Figure 3f).

#### Comparison of WSS Between Feedstocks

**FIGURE 4:**
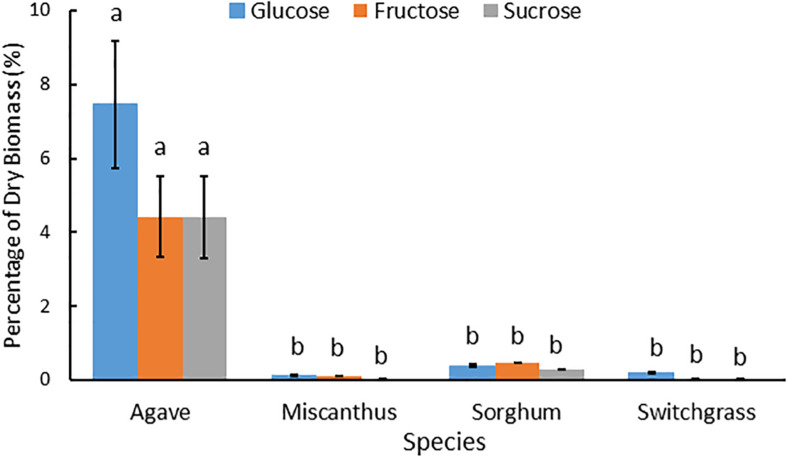
Mean Glc, Fru and Suc as a percentage of dry biomass composition for miscanthus, sorghum, and switchgrass and then all feedstocks including *A. americana* at a different scale to show relative differences. Error bars indicate standard error of the mean (*n* = 16 for *A. americana* and *n* = 3 for grasses). All three sugar concentrations were significantly greater in *A. americana* than in the perennial grasses (*p* < 0.05) from one-way ANOVA and Tukey’s HSD test of differences between species within each sugar type, as indicated by lowercase letters.

Water soluble extracts derived from *A. americana* and the temperate grasses differed by total percent recovered and carbohydrate composition. For *A. americana* measured in this study, water soluble components comprised 52.07% of dry biomass while water soluble compounds only contributed to 6.82, 11.60, and 2.79% of dry biomass in miscanthus, sorghum, and switchgrass, respectively. Water soluble mono- and disaccharides also represented a much larger fraction of total biomass in *A. americana* compared to the temperate grass species (Figure 4). Individual mono- and disaccharide contents greatly differed between the temperate grass species miscanthus, sorghum, and switchgrass (*p* = 0.001), having Glc:Fru:Suc ratios of 6:5:1, 1.3:1.7:1, and 4:1:1, respectively (Figure 4).

#### Structural Polysaccharide Composition in *A. americana*

**FIGURE 5:**
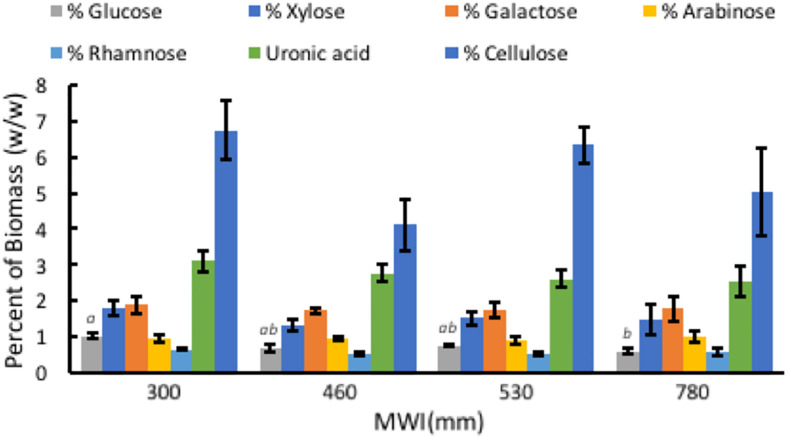
Relationships associated with MWI (mm y^–1^) and polysaccharide composition in dry biomass (%) from TFA hydrolysis and phenol-sulfuric acid hydrolysis of EFBM showing percent glucose, xylose, galactose, arabinose, rhamnose, uronic acid, and cellulose for *A. americana*. Error bars indicate standard error of the mean; *n* = 4. Lowercase letters above bars indicate significant differences in glucose (*p* < 0.05) from one-way ANOVA and Tukey’s HSD test. No significant difference in the other sugars were observed between species (*p* > 0.05).

Composition of *A. americana* leaf biomass was examined to determine the relationship between WUE and structural carbohydrates in the CAM species *A. americana*. Results from TFA hydrolysis of *A. americana* leaf EFBM yielded a mean (*n* = 16) of 5.63% fermentable monosaccharides. There was no significant difference among irrigation treatments in uronic acid content (*p* = 0.59), or monosaccharides liberated by TFA (*p* = 0.54) besides Glc; per one-way ANOVA and Tukey’s HSD test, there was a significant difference (*n* = 4; *p* = 0.0266) between the Glc content in samples from growing conditions with the lowest (300 mm) and highest (780 mm) MWIs (1.0275% ± 0.092% and 0.605% ± 0.094%, respectively) (Supplementary Table 1 and Figure 5). Uronic acids in the form of GalA, a component of pectins HGA and xylogalacturonan, were present in the largest proportion across all treatments (2.76%) compared to other hemicellulose components liberated by TFA hydrolysis.

Phenol-sulfuric acid hydrolysis of the insoluble pellet yielded an average percent cellulose in *A. americana* of 5.55% ± 0.47% (*n* = 16). There was no significant difference in cellulose content across irrigation treatment per one-way ANOVA and Tukey’s HSD test (*p* = 0.168). Cellulose content was 6.74% ± 0.83%, 4.11% ± 0.70%, 6.33% ± 0.50%, 5.01% ± 1.22% for individuals receiving water inputs of 300, 460, 530, and 780 mm y^–1^, respectively (*n* = 4).

#### Comparison of Structural Polysaccharide Composition Between Feedstocks

**FIGURE 6:**
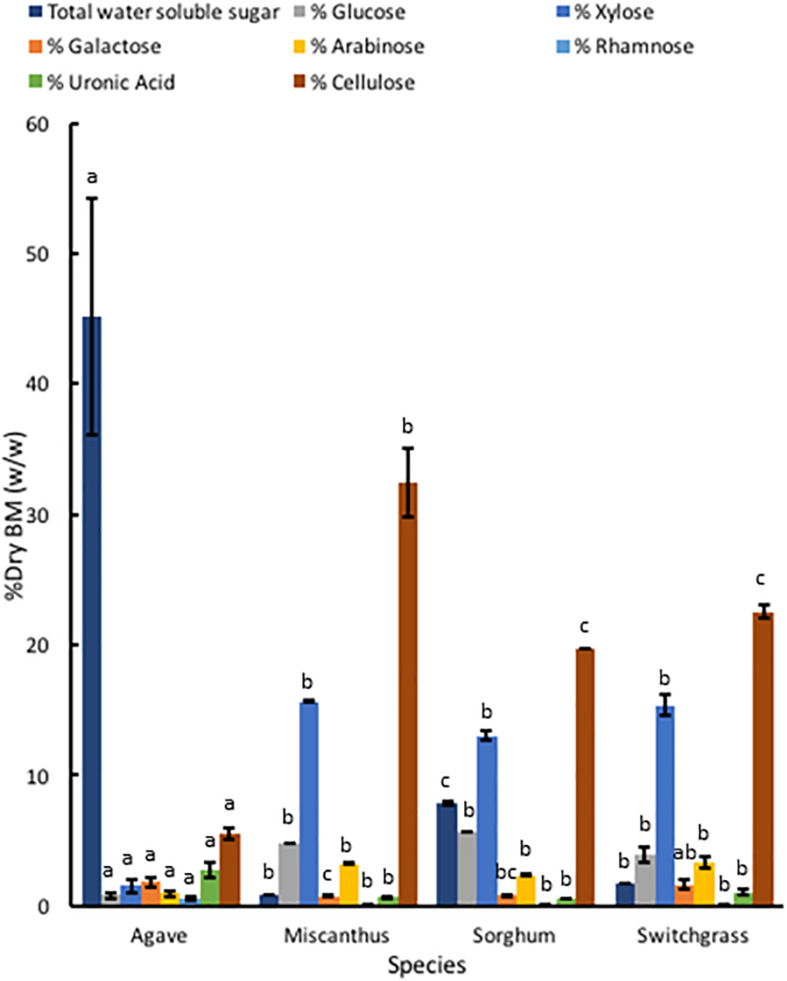
Polysaccharide composition of dry biomass (%) from TFA hydrolysis and phenol-sulfuric acid hydrolysis of EFBM showing mean percent total water-soluble carbohydrates, glucose, xylose, galactose, arabinose, rhamnose, uronic acid, and cellulose for *A. americana*, miscanthus, sorghum, and switchgrass (*n* = 16 for *A. americana* and *n* = 3 for grasses). Lowercase letters above bars indicate significant differences between species (*p* < 0.05) from one-way ANOVA and Tukey’s HSD test of each soluble sugar.

Compared to *A. americana*, the grasses had a larger percentage of dry biomass in structural polysaccharides (Figure 6). TFA hydrolysis of EFBM yielded more total monosaccharides in the grass feedstocks (24.53% ± 0.16%, 21.84% ± 0.32, and 24.22% ± 2.04 for miscanthus, sorghum, and switchgrass, respectively) compared to *A. americana* (5.63% ± 1.23%), however, *A. americana* had a larger mean uronic acid content (2.76% ± 0.59) compared to the other species (0.64% ± 0.05, 0.55% ± 0.03, and 1.04% ± 0.24, respectively) (Figure 6). For all grasses, the largest proportion of monosaccharides from hemicellulose in descending order of abundance were xylose, glucose, arabinose, galactose, and rhamnose. This differs from *A. americana* with galactose as the most abundant fermentable monosaccharide present in hemicellulose (Figure 6). Results from phenol-sulfuric acid hydrolysis of the grasses showed a greater percentage of cellulose contents compared to *A. americana* (*p* = 5.22 × 10^–15^); cellulose content was 32.43% ± 2.67%, 19.66% ± 0.00%, and 22.51% ± 0.50% for miscanthus, sorghum, and switchgrass, respectively, and was 5.55% ± 0.47% for *A. americana* (Figure 6).

### Analysis of GH

**FIGURE 7:**
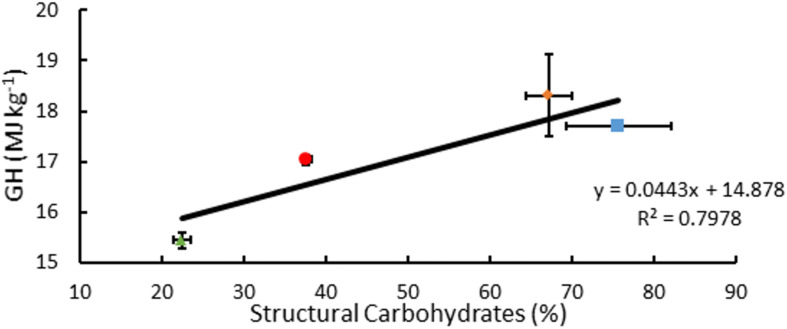
Relationships associated with percentage structural carbohydrates per unit dry biomass (%) and GH for *A. americana* (▲), *Miscanthus* (■), *Sorghum* (∙), and switchgrass (◆). Error bars indicate standard error of the mean; *n* = 3 for GH; n for composition is reported in Supplementary Table 1.

Measured GH of *A. americana* EFBM leaf tissue was 15.44 MJ kg^–1^ ± 0.15 MJ kg^–1^ (*n* = 16), and there was no significant difference between groups receiving differing MWIs as per one-way ANOVA (*p* = 0.096). Of the different feedstocks tested, *A. americana* leaves had the lowest mean measured GH from oxygen bomb calorimetry (15.44 MJ kg^–1^ ± 0.15 MJ kg^–1^; *p* = 0.0065) compared to whole plant tissue samples of miscanthus, sorghum, and switchgrass (Figure 7). Theoretical GH, calculated from equations 2a and 2b, ranged from 17.29 MJ kg^–1^ to 17.63 MJ kg^–1^ for *A. americana*.

### Comparison of Enzymatic Digestibility

**FIGURE 8:**
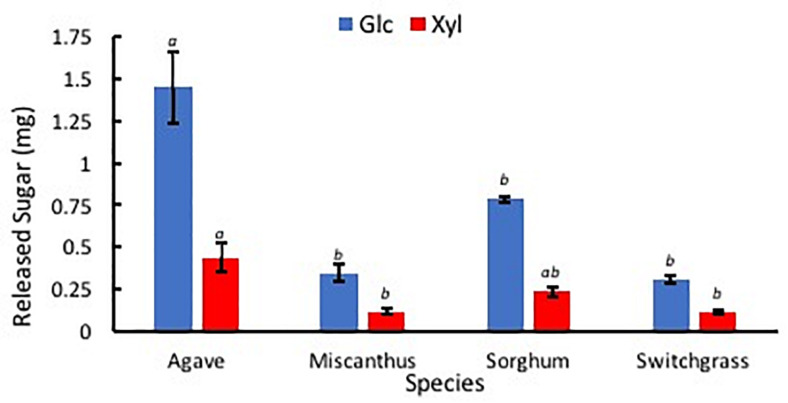
Sugars Glc (blue) and Xyl (red) released from low solids loading enzymatic hydrolysis, expressed in mg, for each of the four species *A. americana* (Agave), miscanthus, sorghum, and switchgrass. Error bars indicate standard error; *n* = 3. Lowercase italic letters indicate significant differences between species by sugar type as per Tukey’s HSD test (*p* < 0.05).

After the hydrolysis treatment, *A. americana* had the greatest amount of sugar products compared to the other feedstocks (*p* = 0.0005); Glc and Xyl released from 0.014 g extractive free biomass was found to be 1.45 mg ± 0.21 mg and 0.44 mg ± 0.09 mg for *A. americana* (*n* = 3; Figure 8). Glc and Xyl released from enzymatic hydrolysis of extractive free biomass from *A. americana* were significantly greater compared to the other feedstocks tested (*p* = 0.00028 and 0.013, respectively), and sorghum had the greatest amount of sugar products compared to the grasses (*p* = 7.67 × 10^–5^; Figure 8).

**FIGURE 9:**
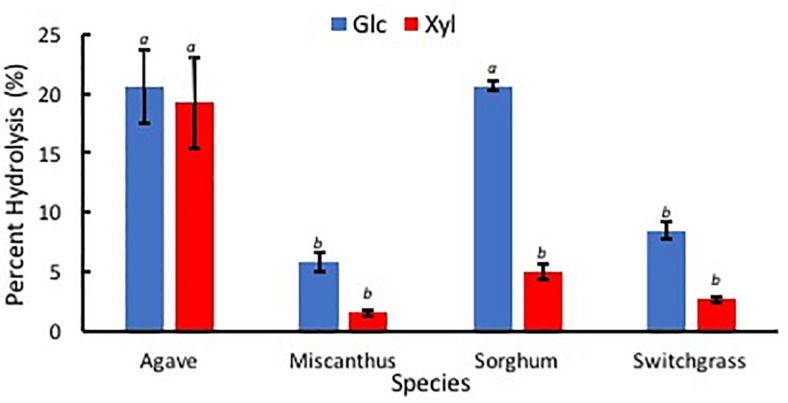
Percent sugars Glc (blue) and Xyl (red) released from low solids loading enzymatic hydrolysis for each of the four species *A. americana* (Agave), miscanthus, sorghum, and switchgrass. Error bars indicate standard error; *n* = 3. Lowercase italic letters indicate significant differences between species by sugar type as per Tukey’s HSD test (*p* < 0.05).

Percent hydrolysis was determined for each Glc and Xyl with respect to contents of each monosaccharide within EFBM. *Agave americana* had the greatest mean percentage of total Glc and Xyl liberated by enzymatic hydrolysis (21.28% ± 3.20%). The percentage of monosaccharides in the other feedstocks, in descending order, were 13.16% ± 0.53% (sorghum), 6.19% ± 0.44% (switchgrass), and 3.93% ± 0.47% (miscanthus). Percent Glc after enzymatic hydrolysis of EFBM was statistically similar in samples from switchgrass and miscanthus (*p* = 0.66), and percent hydrolysis of *A. americana* and sorghum were significantly greater than that of switchgrass and miscanthus as per ANOVA and Tukey’s HSD test (*p* = 0.0017). Glc monomers from *A. americana* (20.58% ± 3.04%) and sorghum (20.64% ± 0.40%) were similar (*p* = 0.90), but more Xyl was released from *A. americana* (19.21% ± 3.77%) compared to sorghum (5.03% ± 0.66%) (*p* = 0.0035; Figure 9). Glc and Xyl released from switchgrass (8.49% ± 0.72% and 2.67% ± 0.28%, respectively) and miscanthus (5.84% ± 0.79% and 1.56% ± 0.22%, respectively) were the lowest of the feedstocks tested (*n* = 3; Figure 9). Percent Xyl released from *A. americana* was significantly greater than that released from the other feedstocks as per ANOVA and Tukey’s HSD test (*p* = 0.0025).

## Discussion

This study evaluated *A. americana* as a potentially less recalcitrant advanced biofuel feedstock to be cultivated in the southwestern United States by analyzing the chemical composition of *A. americana* plants grown in a field trial. Results here indicate that the high proportion of WSS present in *A. americana* leaf tissue can be more easily extracted and fermented even without pretreatment processes to remove hemicelluloses and lignin. Literature values for biomass yield (Grennell, 2014; Arundale et al., 2015; Davis et al., 2016) were used to calculate fuel yield based on WSS and holocellulose content resolved in this study. The results indicate that *A. americana* crops can produce fuel amounts on arid lands that are comparable to fuel yield from temperate grass feedstocks (Table 3).

**TABLE 3:** Biomass yield (dry) and calculated bioethanol (EtOH) yield^a^ expressed in L (ha y)^–1^ for *A. americana (Ag)*, miscanthus (Mi), sorghum (So), and switchgrass (Sw) with respect to water input (mm y^–1^), and water use efficiency (WUE) expressed in kg (ha mm)^–1^ showing fuel yield to water input (Fuel:H_2_O) ratio.

Species	Biomass yield (Mg ha^–1^ y^–1^)	EtOH yield*^*a*^* (L ha^–1^ y^–1^)	Water input (mm y^–1^)	WUE (kg ha^–1^ mm^–1^)	Fuel:H_2_O (L ha^–1^ mm^–1^)
Ag	9.3^1^	6647	530^1^	17.55	12.54
Mi	23.4^2^	8653	1030^2^	22.72	8.40
So	22.0^3^	5998	1019^3^	21.59	5.89
Sw	10.0^2^	3039	1030^2^	10.10	2.95

*^1^Davis et al., 2016; ^2^Arundale et al. (2015); ^3^Grennell (2014). *^*a*^*Ethanol (EtOH) yield calculated from biomass yield reported in first column, summed WSS and holocellulose, 0.51 conversion of sugar to EtOH (w w^–1^), and the density of EtOH (d_*EtOH*_ = 0.789 kg L^–1^) as described in Li et al. (2012).*

Overall, annual biomass yields of *A. americana* are lower compared annual biomass yields of temperate grass feedstocks analyzed in this study (Table 3), but *A. americana* leaf tissue contains WSS that are readily fermentable without pretreatments. *Agave americana* can therefore yield fuel quantities comparable to the temperate grass species even in conditions with low water input because of the large calculated WUE for this species (Table 3). Here, the direct measurements of carbohydrates in *A. americana* indicate biofuel yield from this species would be 6647 L ha^–1^ y^–1^, and the ratio of fuel yield to water input for each of the four species examined in this study was highest for *A. americana* (12.54 L ha^–1^ mm^–1^) (Table 3).

### Composition Analysis

For *A. americana*, experimentally quantified WSS (43.95%), hemicellulose (5.63%), and lignin content (9.06%) were greater than prior literature values, while cellulose (5.55%) and holocellulose content (11.18%) were lower than literature values (Corbin et al., 2015). Miscanthus cellulose content measured in this study was lower than estimates from prior literature (Hodgson et al., 2011; Hayes, 2012; Haffner et al., 2013). Switchgrass had lower water-soluble mono- and disaccharide content in this study relative to prior literature (Samuel et al., 2010 and Yan et al., 2010) that may be due to the short term storage outdoors after harvest, as would be expected in real-world management of the crop. There were comparable percentages of total mono- and disaccharides between miscanthus and switchgrass, while past literature did not report any water-soluble mono- and disaccharides in miscanthus (Samuel et al., 2010; Yan et al., 2010; Hodgson et al., 2011; Hayes, 2012; Haffner et al., 2013; Figure 4). Sorghum composition was also different from literature values (Zhao et al., 2009), as tissue originated from a biomass variety (*i.e.*, CHR-FS4).

This study confirms the findings of past literature that indicate there are high concentrations of WSS within the leaves of *Agave* spp. and other CAM plants (Sánchez-Marroquín and Hope, 1953; Mancilla-Margalli and López, 2006; Borland et al., 2009; Bouaziz et al., 2014; Zacarías-Toledo et al., 2016). Prior collection and analysis of pressed juice from *A. americana* showed leaves contained high concentrations of free glucose (12.7 g L^–1^) (Li et al., 2012). Yet, TFA hydrolysis of the water extract in this study did not yield as much Fru in *A. americana* as would be expected by the large amount of material present in the water extract (Supplementary Table 1). Previous analysis of pressed juice from *A. americana* leaves by Li et al. (2012) showed the presence of unidentified water-soluble oligomeric sugars (4.2 g L^–1^). Past literature has reported high content of uniquely structured fructans (*i.e*., neofructan) present in CAM species, including *A. americana* (Li et al., 2012; Zacarías-Toledo et al., 2016). These neofructans were identified in past studies of *Agave* spp. and are structurally different from inulin isolated from *Chicorium intybus* L., are heterosubstituted and highly branched, and have been shown to have a wholly unique structure, termed agavin, that is more akin to fructans isolated from *Allium* spp. and *Asparagus officinalis* (Mancilla-Margalli and López, 2006; López and Mancilla-Margalli, 2007; Ravenscroft et al., 2009; Arrizon et al., 2010; García-Curbelo et al., 2016). Structural carbohydrates were composed of TFA-soluble hemicelluloses and TFA-insoluble cellulose. The structural fraction of *A. americana* leaf biomass contains mostly cell wall carbohydrates from the residual fiber and cuticle (Li et al., 2012; Pérez-Pimienta et al., 2013). The fiber, which constitutes approximately 25% of dry biomass, is predominately cellulose and contains little lignin compared to other feedstocks of interest (Mylsamy and Rajendran, 2010; Li et al., 2012; Corbin et al., 2015; Yang et al., 2015).

The amount of GalA in *A. americana* tissue indicated that a large proportion of structural pectic oligosaccharide was present within the cell wall (Figure 6). Uronic acids are of interest with respect to biofuel production because they act as inhibitors to fermentation (Jönsson and Martín, 2016). The proportion of rhamnose liberated from TFA hydrolysis was also indicative of a pectic saccharides present in the *A. americana* cell wall which are most abundant in the RG I molecule, however, the smaller proportion of rhamnose compared to the other monosaccharides indicates these may be from RG II, the more substituted pectic polysaccharide that contains a galacturonic acid backbone with highly substituted sidechains. Large proportions of arabinose, galactose, and xylose are indicative of RG I and may be from the associated sidechains containing branched arabinan, unbranched galactan, and type I arabinogalactan (Øbro et al., 2004). The large proportion of arabinose and galactose may also be indicative of arabinogalactan proteins, which contain mostly type II arabinogalactan. The amount of glucose and some of the xylose present in *A. americana* leaf tissue can be attributed to XyG present in non-commelinoid monocot species. However, fucose, which is present in the fucogalacto-XyG, was minimally detectable (values not reported). These results are consistent with *A. americana*, from the order Asparagales, having a Type I cell wall that is rich in pectins and contains XyGs and xylans.

The large amounts of TFA-soluble xylose, glucose, and arabinose that were measured in the grasses are consistent with the presence of xylans and mixed linkage glucans found in commelinoid species; these polymers contain xylan and glucan backbones, respectively, and are largely present in commelinoid monocot species, with mixed linkage glucans occurring uniquely in Poales (Buckeridge et al., 2004). Chromatogram traces of the grasses showed minimal differences between them except miscanthus had an unidentified peak *ca.* 21 min and had a lower response for rhamnose compared to galactose (Supplementary Figure 2). The proportion of arabinose to xylose is consistent with the presence of arabinoxylan. Uronic acid was present as GalA in all three temperate grass species but was a much lower proportion of total hemicellulosic material compared to *A. americana*, which is consistent with the grasses having a pectin-poor cell wall matrix. It is well-known that grasses have Type II cell walls.

### Total Carbohydrates, WUE and Fuel Yield

Due to the greater WSS and greater WUE, the fuel yield from *A. americana* per unit of water input is greater than the fuel yield from the other feedstocks per unit of water input (Table 3). In the first field trial examining *A. americana* productivity on arid lands, Davis et al. (2016) found dry biomass yield to be 2.5–9.3 Mg (ha y) ^–1^ with maximum productivity and WUE [17.55 kg ha^–1^ mm water (H_2_O^–1^)] with 530 mm H_2_O input. Yan et al. (2011) showed bioethanol derived from *A. tequilana* has a greater GHG offset compared to maize and switchgrass feedstocks, and *A. tequilana* may produce more ethanol per unit area, compared to maize and switchgrass, when grown under favorable conditions. However, this study is the first to examine how productivity and associated WUE of field grown *A. americana* relates to the concentration of fermentable carbohydrates and final biofuel yield.

Crassulacean Acid Metabolism physiology has a principle advantage over C_3_ and C_4_ photosynthesis because nocturnal carbon assimilation by the enzyme phosphoenolpyruvate carboxylase (PEPC), activity that is temporally separated from the daytime activity of the enzyme ribulose-1,5-bisphosphate carboxylase/oxygenase (RUBISCO), leads to lessened evapotranspiration and greater WUE (Nobel, 1991, 2003; Kluge and Ting, 2012). The WUE of CAM species has been observed to be 4- to 8-fold higher than C_3_ plants (Neales, 1973; Nobel, 1991, 2003; Kluge and Ting, 2012) and CAM species can therefore be more productive than C_3_ and C_4_ plant species in drought conditions (Ehrler, 1983; Cushman et al., 2015; Davis S.C. et al., 2015; Yang et al., 2015). Many CAM species can avoid hydraulic limitations due to adaptations (*e.g*. drought induced abscission of roots/leaves, succulence, rosette morphology, sunken stomata, decreased stomatal density) that allow them to maintain turgor even in a xeric environment (Woerner and Martin, 1999; Nobel, 2003; Borland et al., 2009). *Agave* spp. can resume physiological function after long periods of drought by quickly achieving comparable transpiration rates when water becomes available (Ehrler, 1983).

*Agave* spp. also exhibit high photosynthetic productivity across a range of environmental conditions (Neales, 1973; Gentry, 1982; Nobel, 2003; García-Moya et al., 2011). In Australia and the semi-arid southwestern US, field trials have been established to test *Agave* spp. as potential biofuel feedstocks (Chambers et al., 2010; Holtum et al., 2011; Davis et al., 2016). Li et al. (2012) compared theoretical maximum ethanol yields derived from *A. americana*, poplar and switchgrass cropping systems, and projected bioethanol yields of 3645–12390, 4819, and 5311 L ha^–1^ y^–1^, respectively. Davis et al. (2014) found biofuel yields for *A. fourcroydes*, *A. tequilana* and *A. sisalana* to be 3300, 9700, and 4700 L ha^–1^ y^–1^.

### Energy Content of Biomass

Gross heat of combustion was measured in an oxygen bomb calorimeter and expressed in MJ kg^–1^ to determine total recoverable energy from EFBM for each of the four crop species (*A. americana*, miscanthus, sorghum, and switchgrass). Literature has previously shown GH values to be highly correlated with lignin and extractives values (Demirbaş, 2001), but results from this study contradict that finding in the case of *A. americana*. In the case of sorghum and switchgrass, equations 2a and 2b accurately predicted the range of measured GH value, however in the case of *A. americana* and miscanthus, results were overestimated by 11.30 and 1.74%, respectively, compared to the lower of the calculated theoretical GH values for that species. Notably, *A. americana* had a measured GH value that was less than the y-intercept of both equations 2a and 2b. Similarly low values for GH have been reported in the literature for CAM species like *A. tequilana* and *O. ficus-indica*, with GH of 17.50 and 16.95 MJ kg^–1^, respectively (Yang et al., 2015).

Composition of biomass as reported in previous literature for species examined within this study contradict the correlation of GH with% lignin, in the case of *A. americana*. From composition data compiled from literature values (Thammasouk et al., 1997; Zhao et al., 2009; Yan et al., 2010; Hodgson et al., 2011; Hayes, 2012; Haffner et al., 2013; Pérez-Pimienta et al., 2013; Corbin et al., 2015), GH was found to be weakly correlated with percent lignin (*R*^2^ = 0.516) but was found to be more correlated with percent structural carbohydrates (*R*^2^ = 0.798) (Figure 7). Friedl et al. (2005) propose that GH is best correlated with elemental composition analysis with respect to carbon (C), hydrogen (H), and nitrogen (N) contents. These results indicate that estimating GH using algebraic models that solely rely on lignin may not be appropriate for some species, including *A. americana* where lignin content may not be related to the biomass recalcitrance during enzymatic digestion.

### Comparison of Enzymatic Digestibility

It was hypothesized that *A. americana* will be more susceptible to enzymatic hydrolysis and produce a higher-quality hydrolysate with a higher concentration of WSS compared to other candidate feedstocks because of the lower percentage of lignin; lignin and hydrolyzed residues are known to inhibit fermentation and enzymatic hydrolysis (Berlin et al., 2006; Ximenes et al., 2010). The results of this study confirmed that *A. americana* was most susceptible to enzymatic hydrolysis compared to the other feedstocks tested.

## Conclusion

Despite the lower overall energy content of *A. americana* relative to the advanced cellulosic C_4_ crops, potential liquid fuel yields from *A. americana* are greater because fermentable sugars are more easily produced from biomass feedstocks. Total amount of sugars liberated from enzymatic hydrolysis of *A. americana* were almost double that of sorghum, more than triple that of switchgrass, and more than five-fold that of miscanthus. These results suggest that *A. americana* is a less recalcitrant feedstock, and a lower quantity of enzymes are necessary to achieve comparable depolymerization of structural polysaccharides to fermentable monosaccharides than other lignocellulosic feedstocks. Results from comparison of the four feedstocks indicated that *A. americana* is the most easily hydrolyzed relative to sorghum, switchgrass, and miscanthus.

## Data Availability statement

All datasets generated for this study are included in the article/Supplementary Material. Raw datasets are available from authors by request.

## Author Contributions

AJ, MH, and SD designed the study and research objectives. AJ collected biomass, calculated yield data, and performed statistical analysis. AJ and YZ processed biomass samples. AJ, YZ, SD, and MH conducted laboratory experiments and data analysis and contributed section content and edits to subsequent drafts. AJ and SD drafted the initial manuscript. SD and MH financially supported the project work and manuscript publication.

## Conflict of Interest

The authors declare that the research was conducted in the absence of any commercial or financial relationships that could be construed as a potential conflict of interest.
