# Supplementary material for: Tissue Composition of Agave americana L. Yields Greater Carbohydrates From Enzymatic Hydrolysis Than Advanced Bioenergy Crops
Source: Front Plant Sci. 2020 Jun 11;11:654. doi: 10.3389/fpls.2020.00654 (PMC7300260; doi:10.3389/fpls.2020.00654)
Supplement: Supplementary file 1 [file Data_Sheet_1.PDF]

Supplemental Information

Supplemental Table 1

Composition of A. americana leaf tissue per mean annual water input (MWI; mm y^{-1}) showing component biomass as percent dry mass (w w^{-1}) with range indicating standard error (n = 4) between treatments, root mean square (RMS), and standard deviation (stdev) across all samples (N = 16). Lowercase italicized letter indicates significant difference (P < 0.05) as per ANOVA and Tukey's HSD test.

		% Dry biomass (w w^{-1})	MWI (mm y^{-1})				RMS	stdev
			300	460	530	780		
		%Soil	5.92 ± 0.71	9.68 ± 0.86	8.18 ± 1.05	8.27 ± 1.25	8.01	2.25
Water-Soluble	Mono- and disaccharides	%Glc	7.26 ± 1.33	6.89 ± 0.14	8.71 ± 0.94	6.98 ± 0.54	7.46	1.72
		%Fru	4.38 ± 0.73	4.39 ± 0.50	4.89 ± 0.74	4.02 ± 0.17	4.42	1.09
		%Suc	4.99 ± 0.57	3.64 ± 0.24	5.00 ± 0.61	4.07 ± 0.57	4.42	1.11
	Oligosaccharides	%Glc (oligo)	2.83 ± 0.73	4.56 ± 0.45	3.18 ± 0.33	4.22 ± 0.76	3.70	1.30
		%Fru (oligo)	3.45 ± 0.45***a***	6.19 ± 0.61***b***	3.06 ± 0.30***a***	5.28 ± 0.34***ab***	4.49	1.55
		%Suc (oligo)	0.23 ± 0.23	0.0 ± 0.0	0.08 ± 0.08	0.02 ± 0.01	0.08	0.24
		%Other	16.06 ± 3.65	21.35 ± 6.37	16.31 ± 4.20	23.79 ± 2.33	19.38	8.56

	%Water Extr. Others	14.72 ± 3.94	13.56 ± 4.36	14.78 ± 2.91	16.44 ± 4.86	14.88	7.38
	%Ethanol Extr.	1.97 ± 0.60	3.23 ± 0.12	3.81 ± 0.51	1.78 ± 0.65	2.70	1.27
	%Struc. Protein	2.72 ± 0.12	2.35 ± 0.44	2.93 ± 0.86	2.06 ± 0.39	2.51	1.00
	%Struc. Inorg.	5.69 ± 0.69	4.75 ± 0.48	4.42 ± 0.50	6.29 ± 1.03	5.29	1.49
	%Lignin	9.66 ± 0.62	9.75 ± 0.93	8.34 ±1.24	8.51 ± 0.62	9.06	1.73
TFA-soluble	%Glc	1.03 ± 0.09***a***	0.70 ± 0.11***ab***	0.76 ± 0.03***ab***	0.61 ± 0.09***b***	0.77	0.22
	%Xyl	1.81 ± 0.19	1.32 ± 0.17	1.51 ± 0.17	1.49 ± 0.45	1.53	0.52
	%Gal	1.89 ± 0.25	1.73 ± 0.09	1.75 ± 0.19	1.78 ± 0.33	1.79	0.42
	%Ara	0.98 ± 0.11	0.94 ± 0.05	0.91 ± 0.10	1.01 ± 0.16	0.96	0.21
	%Rha	0.66 ± 0.04	0.55 ± 0.05	0.54 ± 0.06	0.59 ± 0.09	0.58	0.13
	%GalA	3.11 ± 0.28	2.77 ± 0.24	2.61 ± 0.23	2.55 ± 0.43	2.76	0.59

%Cellulose	6.75 ± 0.83	4.11 ± 0.70	6.33 ± 0.50	5.01 ± 1.22	5.55	0.47
**Total %**	**96.08 ± 1.36**	**102.45 ± 1.94**	**98.09 ± 2.27**	**104.74 ± 4.54**	**100.3**	**6.14**

Supplemental Figure legends:

Supplemental *Figure 1*: Map of lignocellulosic grass plots in Ohio University Ridges Land Lab showing managed plantings of miscanthus (Mi), sorghum (So), and switchgrass (Sw); each plot measures 10 m x 10 m with 2 m spacing between plots.

Supplemental *Figure 2*. HPAEC-PAD chromatogram traces from TFA hydrolysate from EFBM showing relative response for fucose (3.42min), rhamnose (6.98min), arabinose (7.50min), galactose (9.70min), xylose (13.10min), and galacturonic acid (26.85min), for (A) *A. americana*, (B) miscanthus, (C) sorghum, and (D) switchgrass.

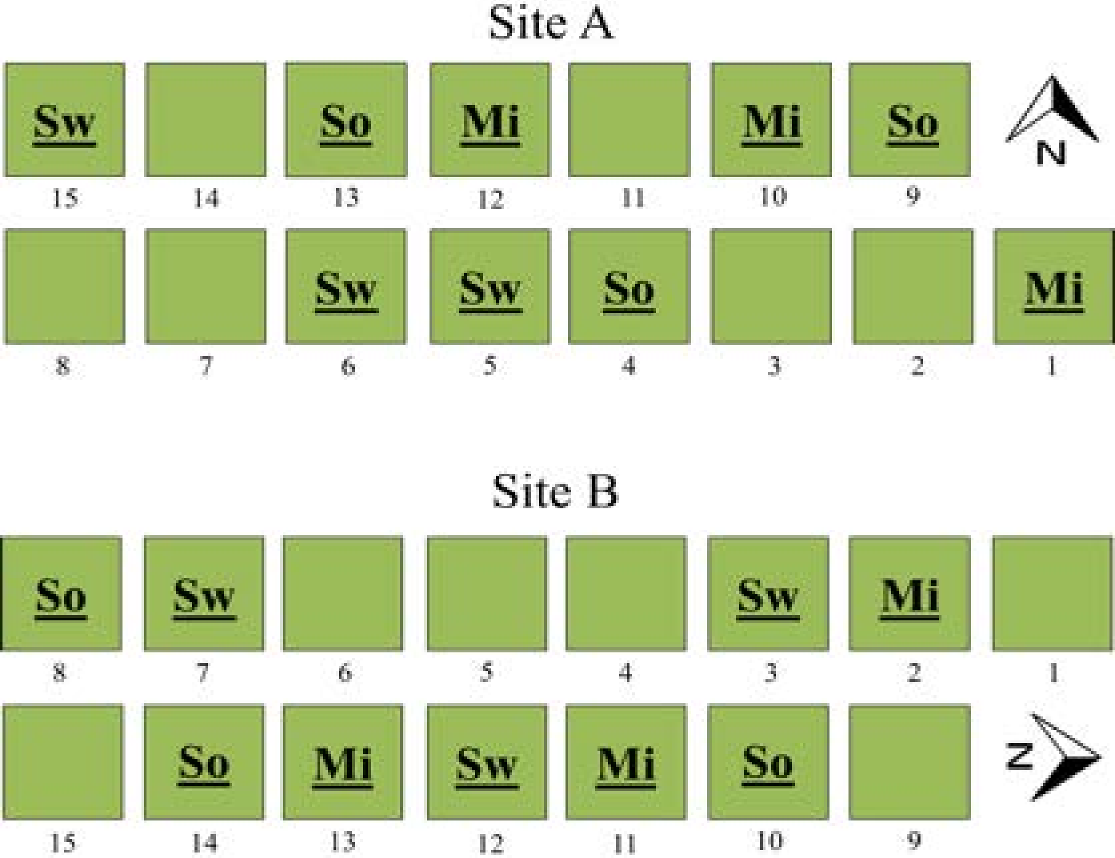
Site A
Sw
So
Mi
Mi
So
N
15
14
13
12
11
10
9
Sw
Sw
So
Mi
8
7
6
5
4
3
2
1
Site B
So
Sw
Sw
Mi
8
7
6
5
4
3
2
1
So
Mi
Sw
Mi
So
N
15
14
13
12
11
10
9

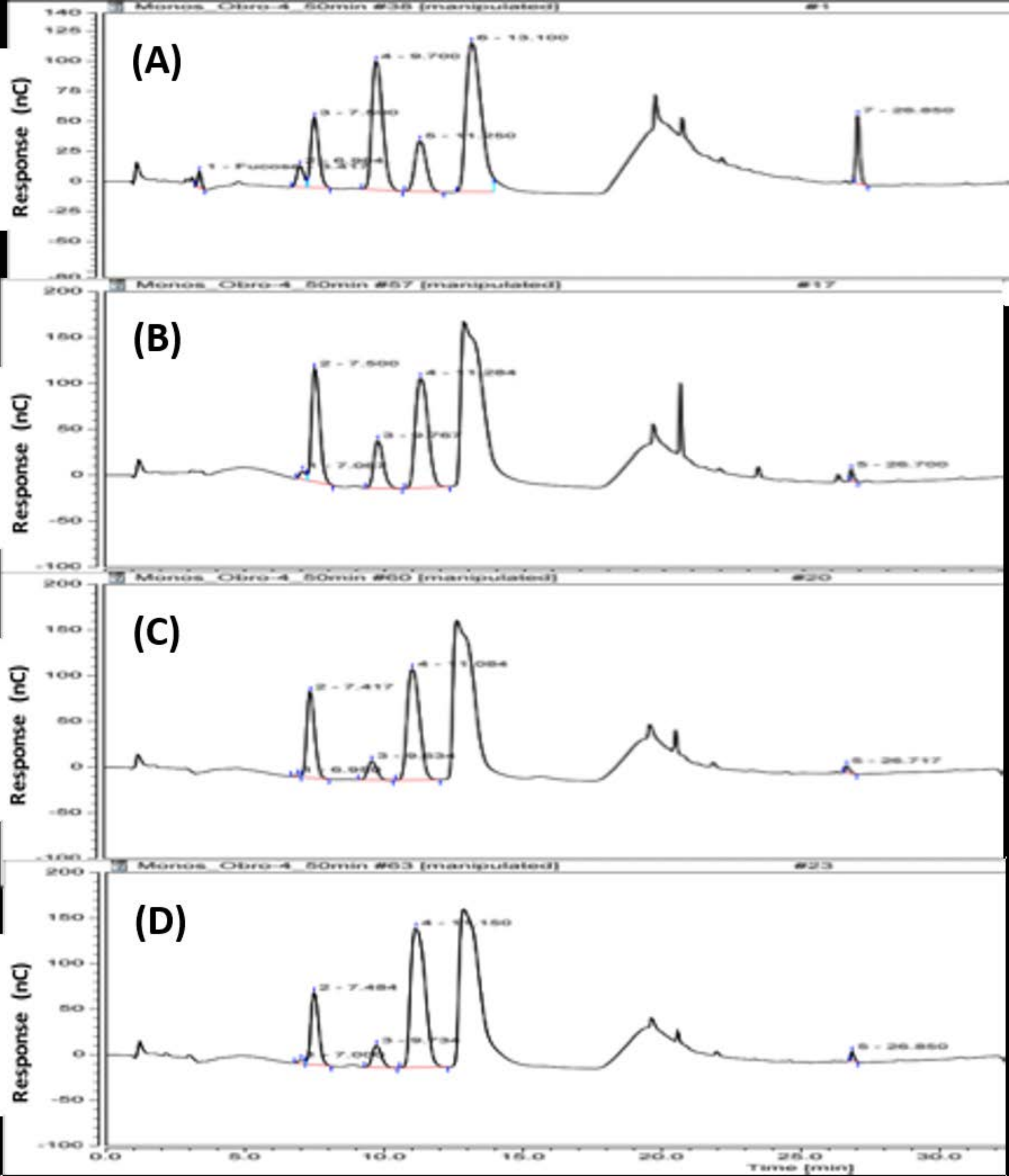
Monos_Obro-4_50min #38 (manipulated)
#1
(A)
Response (nC)
1 - Fucose
4 - 9.700
5 - 11.250
6 - 13.100
7 - 26.850
Monos_Obro-4_50min #57 (manipulated)
#17
(B)
Response (nC)
2 - 7.500
3 - 9.767
4 - 11.284
5 - 26.700
Monos_Obro-4_50min #60 (manipulated)
#20
(C)
Response (nC)
2 - 7.417
4 - 11.084
5 - 26.717
Monos_Obro-4_50min #63 (manipulated)
#23
(D)
Response (nC)
2 - 7.484
5 - 26.850
Time [min]
